# Antifungal Activity against Filamentous Fungi of Ts1, a Multifunctional Toxin from *Tityus serrulatus* Scorpion Venom

**DOI:** 10.3389/fmicb.2017.00984

**Published:** 2017-06-06

**Authors:** Welligton M. Santussi, Karla C. F. Bordon, Ana P. N. Rodrigues Alves, Camila T. Cologna, Suraia Said, Eliane C. Arantes

**Affiliations:** ^1^Laboratory of Animal Toxins, Department of Physics and Chemistry, School of Pharmaceutical Sciences of Ribeirão Preto, University of São PauloRibeirão Preto, Brazil; ^2^Laboratory of Industrial Enzymology, Department of Pharmaceutical Sciences, School of Pharmaceutical Sciences of Ribeirão Preto, University of São PauloRibeirão Preto, Brazil

**Keywords:** *Tityus serrulatus*, defensins, fungi, antifungal drugs, cysteine-rich antifungal proteins, antimicrobial peptides, antimycotic, drosomycin

## Abstract

Antimicrobial peptides (AMPs) are ubiquitous and multipotent components of the innate immune defense arsenal used by both prokaryotic and eukaryotic organisms. The search for new AMPs has increased in recent years, due to the growing development of microbial resistance to therapeutical drugs. In this work, we evaluate the effects of *Tityus serrulatus* venom (Tsv), its fractions and its major toxin Ts1, a beta-neurotoxin, on fungi growth. The fractions were obtained by ion-exchange chromatography of Tsv. The growth inhibition of 11 pathogenic and non-pathogenic filamentous fungi (*Aspergillus fumigatus, A. nidulans, A. niger, A. terreus, Neurospora crassa, Penicillium corylophilum, P. ochrochloron, P. verrucosum, P. viridicatum, P. waksmanii*, and *Talaromyces flavus*) was evaluated by quantitative microplate reader assay. Tsv (100 and 500 μg/well, which correspond to 1 and 5 mg/mL, respectively, of total soluble protein) was active in inhibiting growth of *A. nidulans, A. terreus, P. corylophilum*, and *P. verrucosum*, especially in the higher concentration used and at the first 30 h. After this period, fungi might have used Tsv components as alternative sources of nutrients, and therefore, increased their growth tax. Only fractions IX, X, XI, XIIA, XIIB (3 and 7.5 μg/well, which correspond to 30 and 75 μg/mL, respectively, of total soluble protein) and Ts1 (1.5, 3, and 6 μg/well, which correspond to 2.18, 4.36, and 8.72 μM, respectively) showed antifungal activity. Ts1 showed to be a non-morphogenic toxin with dose-dependent activity against *A. nidulans*, inhibiting 100% of fungal growth from 3 μg/well (4.36 μM). The inhibitory effect of Ts1 against *A. nidulans* growth was accompanied by fungistatic effects and was not amended by 1 mM CaCl_2_ or tetrodotoxin (46.98 and 93.96 μM). The structural differences between Ts1 and drosomycin, a potent cysteine-rich antifungal peptide, are discussed here. Our results highlight the antifungal potential of the first cysteine-containing scorpion toxin. Since Ts1 is a multifunctional toxin, we suggest that it could be used as a template in the design of engineered scorpion AMPs and in the search for new mechanisms of action of antifungal drugs.

## Introduction

Scorpion venoms are a rich source of peptide toxins, insoluble mucus, bioactive amines, hypotensins, proteinases, hyaluronidase, bradykinin-potentiating peptide, kallikrein inhibitor, allergenic proteins, and other peptides whose biological functions are still not clarified (Bordon et al., 2015). Their best studied toxins interact specifically with voltage-gated Na^+^ and K^+^ channels and are toxic both to vertebrates and invertebrates. Na^+^ channel toxins have 61–76 amino acid residues, stabilized by four disulfide bonds, whereas the K^+^ channel toxins present 22–47 amino acid residues, stabilized by three disulfide bonds (Becerril et al., 1997; Tytgat et al., 1999; Lewis and Garcia, 2003; Rodríguez de la Vega and Possani, 2004, 2005).

In the course of the evolution process, some arthropods such as scorpions have evolved, despite the others components, AMPs in their venom (Kuhn-Nentwig et al., 2004). The presence of AMPs in scorpion venom might be related to the protection of the venom gland against infection and also to facilitate the action of neurotoxins (Harrison et al., 2014). AMPs are pervasive and evolutionarily ancient tools of host innate defense against pathogens. They are widespread in nature and are found in bacteria, protozoa, plants, insects, fungi and mammals (Hegedus and Marx, 2013). Some of them are known as small cationic antimicrobial peptides (SCAMPs) or defensins (Zhu and Tytgat, 2004). Those peptides generally adopt an amphipathic structure with cationic and hydrophobic properties which may have a role in the interaction with the cell membranes of microorganism. Amphipathic α-helical AMPs isolated from scorpion venoms function as part of the innate defense mechanism against different kinds of pathogens (Zasloff, 2002). Of the impressive number of antimicrobial structures reported, at least 50% were identified in invertebrates (Bulet et al., 1999).

There is a great variation in function, structure, sensible organisms, expression pattern and origin of AMPs, what make their classification difficult and somewhat arbitrary so far (Hegedus and Marx, 2013). In spite of great differences in size and amino acid composition, most of the AMPs can be classified into 3 groups according to its structural characteristics: (1) lacking cysteine alpha-helical linear peptides, also known as non-disulfide-bridged peptides (NDBPs), (2) cysteine-containing peptides with disulfide bonds and (3) peptides with an over-representation of proline, histidine, tryptophan, or glycine residues (Bulet et al., 2004; Zhu and Tytgat, 2004; Harrison et al., 2014). To this date, over 40 scorpion AMPs have been characterized and classified among the first and second AMP classes, without any representatives on the third one (Harrison et al., 2016) (for review, see Harrison et al., 2014).

In regards to the second group, the first scorpion cysteine-containing AMP was isolated from the *Pandinus imperator* venom and it was named scorpine (Conde et al., 2000). Scorpine was assayed against gram positive (*Bacillus subtilis*) and gram negative (*Klebsiella pneumonia*) bacteria and was active against both (minimal inhibitory concentration – MIC – 1–10 μM). This toxin has a unique structure with a molecular mass of 8.3 kDa stabilized by three disulfide bonds. Its *N*-terminal presents similarities with cecropins, while the *C*-terminal shows similarities with scorpions’ defensins (Conde et al., 2000). On the following years, others scorpine-like toxins were isolated from the scorpions *Opistophtalmus carinatus* (opiscorpine 1–4), *Heterometrus laoticus* (heteroscorpine-1), *Hadrurus gertschi* (Hge-scorpine), *Opisthacanthus cayaporum* (Ocy39.87), and *Urodacus yaschenkoi* (UySCl 1-2) (Zhu and Tytgat, 2004; Uawonggul et al., 2007; Diego-Garcia et al., 2008; Schwartz et al., 2008; Luna-Ramirez et al., 2013) and expressed from the venom cDNA library of scorpion *Euscorpiops validus* (scorpine-like peptide Ev37) (Feng et al., 2013).

Remarkably, six AMPs from *T. discrepans* venom were described and named bactridines (Bacts 1-6). Those molecules are hydrophilic, non-amphipathic and positively charged polypeptides with four disulfide bonds stabilizing a chain of more than 60 amino acids (Diaz et al., 2009). Those peptides, especially Bact 2, have aroused the attention of scientific community but not due to their antimicrobial activity, which is not potent when compared to others AMPs. The spotlights put on Bact 2 rise on its activity as a β-modulator of Na^+^ channel. Bactridines were the first group of scorpion toxins to combine those two functions (Peigneur et al., 2012; Harrison et al., 2014).

Despite the in depth studies concerning the antibacterial activity of AMPs from group 2, studies regarding the antifungal activity of those toxins remain limited. Drosomycin was the first antifungal peptide isolated from insects and is comprised of 44 amino acid residues linked by four disulfide bonds (Michaut et al., 1996). Recently, a cysteine-containing AMP from the *Avicularia juruensis* spider venom, designated as juruin, showed antifungal activity against *Candida* strains and *Aspergillus niger* (Ayroza et al., 2012). Antifungal activity was also described for pandinin-2 from *P. imperator* venom (Corzo et al., 2001) and for seven synthetic peptides which had their primary sequence obtained through transcriptome analysis of *T. obscurus, H. gertschi*, and *O. cayaporum* venom glands (Guilhelmelli et al., 2016); however, all of them are NDBPs.

The fact that fungi are eukaryotes makes difficult the development of drugs that inhibit fungal growth, without a consequent toxicity to the host (Stephenson, 1997). Fungi are capable of causing superficial or severe, often lethal, invasive infections in humans, mainly in immunocompromised patients (transplant recipients under immunosuppressive therapy, cancer patients treated with cytotoxic drugs, and AIDS patients) who are highly susceptible to fungal infections (Ortiz et al., 2015). Aspergillosis is the most frequent fungal infection in severally ill patients and its prevalence has increased in the latest years (Vincent et al., 2009; Tortorano et al., 2012; Cabezas-Quintario et al., 2016). Moreover, increasing antifungal resistance has been reported for the existing antifungal agents. Some fungi are inherently resistant and the biofilm mode of fungal growth is highly resistant to the available drugs (Seneviratne and Rosa, 2016). Thereby, the search for new antifungal agents is urgently required.

Based on that, this study shows the first antifungal screening of *T. serrulatus* venom (Tsv), Tsv fractions and Ts1, the major component of this venom, against 11 different pathogenic and non-pathogenic filamentous fungi: *A. fumigatus, A. nidulans, A. niger, A. terreus, Neurospora crassa, Penicillium corylophilum, P. ochrochloron, P. verrucosum, P. viridicatum, P. waksmanii*, and *Talaromyces flavus*. *A. fumigatus* is the main cause of opportunistic fungal infections in humans (Brakhage, 2005), while *A. terreus* causes superficial and invasive mycosis (Kniemeyer, 2011). The other evaluated fungal species of the genus *Aspergillus* play significant roles as model organisms in basic research and for manufacturing of a wide range of commercial enzymes (Pontecorvo et al., 1953; Baddley et al., 2003). *T. flavus* (formerly known as *P. vermiculatum*) and the genus *Penicillium* have been reported to be producers of secondary metabolites with antimicrobial, antiprotozoal, antitumoral, or insecticidal activities (Freitas et al., 2002; Elias et al., 2006). *N. crassa* is able to degrade phenolic pollutants (Luke and Burton, 2001), and *P. corylophilum* is a plant pathogen and a parasite of fish (Gozlan et al., 2014).

## Materials and Methods

### Soluble Venom

*Tityus serrulatus* venom was obtained by electrical stimulation (Lowe and Farrell, 2011) of scorpions maintained at the vivarium “Biotério Central” (School of Medicine of Ribeirão Preto, University of São Paulo, Brazil), in accordance with the guidelines of the Brazilian College for Animal Experimentation (COBEA) and Ibama, Brazilian Institute of Environment. Desiccated Tsv was suspended in 0.9% (m/V) NaCl and centrifuged at 11,270 × *g*, 25°C, for 5 min. The protein concentration of the soluble Tsv and Ts1 was estimated at 280 nm by a spectrophotometer (U-2001, Hitachi Instrument Inc., Tokyo, Japan) using the extinction coefficient (${𝜀}_{\mathrm{280nm}}^{1mg/mL}$) of 1.65 for the soluble Tsv (Pucca et al., 2014) and 3.548 for the pure Ts1^1^. The protein concentration of the soluble Tsv fractions was estimated by 280/205 nm absorption method (Scopes, 1974).

### Purification Procedure

Desiccated Tsv was fractionated on a CM-cellulose-52 column, resulting in the fractions designated as I to XIII. The eluted fraction XIII was rechromatographed on a C18 reversed-phase (C18-RP) column (Shim Pack CLC-ODS 5 μm, 0.46 cm × 25.0 cm, Shimadzu Instruments Corp., Tokyo, Japan) using a fast protein liquid chromatography (FPLC) system and pure Ts1 was obtained, as previously described (Vasconcelos et al., 2005). The homogeneity of the samples was analyzed by Polyacrylamide Gel Electrophoresis (PAGE, 10%) carried out according to Reisfeld et al. (1962) with modifications described by Arantes et al. (1989). The fractions I to XII-B eluted from the cationic chromatography and pure Ts1 (C18-RP-FPLC) were used in the subsequent assays.

### Fungi

*Aspergillus nidulans* strain FGSC-A26 *(biA1, veA1)* and *N*. *crassa* (74 OR 8-1a, ATCC) were acquired from the Fungal Genetics Stock Center and American Type Culture Collection, respectively. The other fungi were isolated from Brazilian soil samples by Dr. Said S., identified and deposited in the collection of “Fundação André Tosello de Pesquisa e Tecnologia”^2^ (cct@fat.org.br) and cataloged as follows: A. fumigatus (CCT 7168), *A. niger* (CCT 7653), *A. terreus* (CCT 7640), *P. corylophilum* (CCT 7679), *P. ochrochloron* (CCT 7672), *P. verrucosum* (CCT 7680), *P. viridicatum* (CCT 7681), *P. waksmanii* (CCT 7684), and *T. flavus* (CCT 7682). Fungi cultures were maintained on PDA slants and preserved at 4°C with sub-culturing in regular intervals. Seven-days-old cultures (derived from the original stock cultures) grown on PDA tubes at 30°C were used as inoculum.

### Antifungal Activity Assay

The microplate reader assay for fungal growth inhibition was done as previously described by Broekaert et al. (1990) with some modifications. For the preparation of fungi culture, inoculums were transferred to tubes containing PDA and allowed to grow at 30°C in the dark for 7 days. The harvest of spores was carried out flooding spores with sterile water and rubbing with a sterile spatula. The suspensions were then filtered through three layers of muslin. Spores were quantified in a Neubauer chamber for appropriate dilutions. The suspensions were diluted until reach the density of 2 × 10^4^ spores/mL.

**FIGURE 1:**
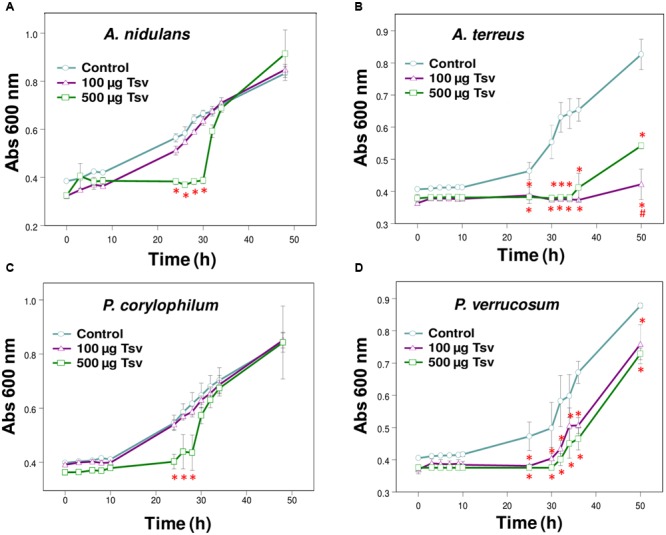
Effect of Tsv on fungal growth. Fungal growth was monitored by measuring the absorbance of fungal culture at 600 nm during 48 h, at different intervals of time. Control (open circle, no venom), Tsv 100 μg/well (open triangle) and 500 μg/well (open square). **(A)** *A. nidulans*; **(B)** *A. terreus*; **(C)** *P. corylophilum*; **(D)** *P. verrucosum*. Each point represents the mean ± SD (*n* = 4) of the absorbance of fungal culture at each time. ^∗^*p* < 0.05 compared to the control, ^#^*p* < 0.01 compared to the other experimental group (one-way ANOVA).

**FIGURE 2:**
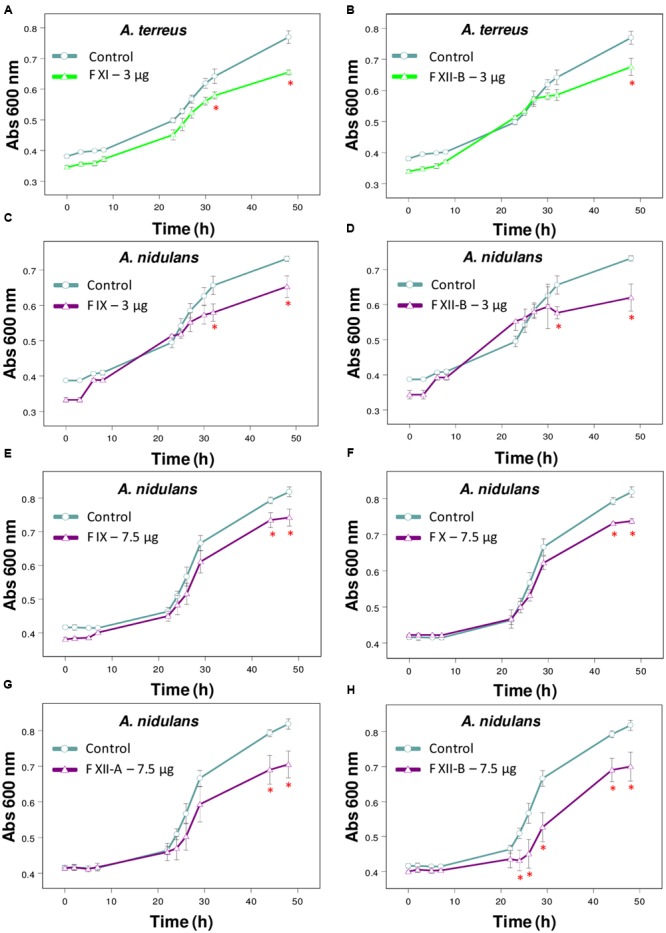
Effect of Tsv fractions on fungal growth. **(A,B)** *A. terreus* using 3 μg/well of fractions **(A)** XI and **(B)** XII-B. **(C,D**) *A. nidulans* using 3 μg/well or **(E–H)** 7.5 μg/well of fractions. **(C,E)** IX, **(D,H)** XII-B, **(F)** X, and **(G)** XII-A. Fungal growth was monitored by measuring the absorbance of fungal culture at 600 nm during 48 h, at different intervals of time. Control (open circle, no venom), fractions (open triangle). Each point represents the mean ± SD (*n* = 4) of the absorbance of fungal culture at each time. ^∗^*p* < 0.05 compared to the control (one-way ANOVA).

**FIGURE 3:**
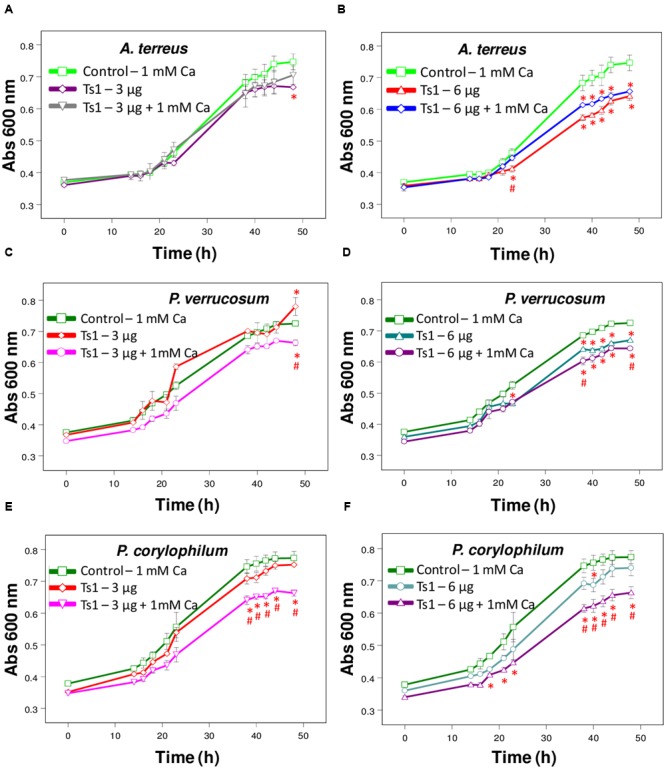
Effect of Ts1 on fungal growth. Control (open circle, 1 mM Ca^2+^, no venom), **(A,C,E)** Ts1 (3 μg/well) or (**B,D,F**; 6 μg/well), in the presence or absence of 1 mM Ca^2+^ were evaluated on **(A,B)** *A. terreus* **(C,D)**, *P. verrucosum*, and **(E,F)** *P. corylophilum* growth. Fungal growth was monitored by measuring the absorbance of fungal culture at 600 nm during 48 h, at different intervals of time. Each point represents the mean ± SD (*n* = 4) of the absorbance of fungal culture at each time. ^∗^*p* < 0.05 compared to the control, ^#^*p* < 0.01 compared to the other experimental group (one-way ANOVA).

**FIGURE 4:**
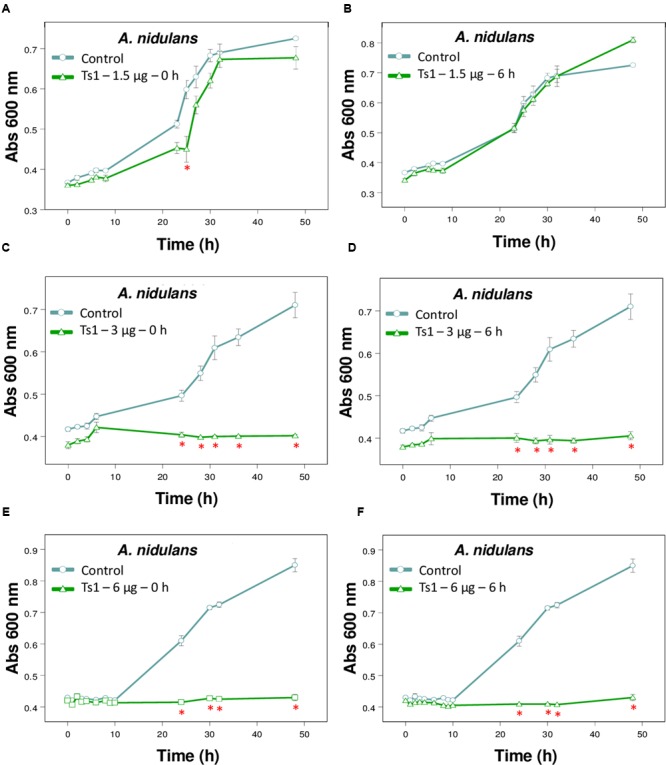
Effect of different amounts of Ts1 on *Aspergillus nidulans* growth at different addition time. Ts1 (open triangle; **A,B**) 1.5 μg/well, **(C,D)** 3 μg/well or **(E,F)** 6 μg/well was added to culture medium **(A,C,E)** at zero time or **(B,D,F)** after 6 h. Control (open circle, no toxin). Fungal growth was monitored by measuring the absorbance of fungal culture at 600 nm during 48 h, at different intervals of time. Each point represents the mean ± SD (*n* = 4) of the absorbance of fungal culture at each time. ^∗^*p* < 0.05 compared to the control (one-way ANOVA).

**FIGURE 5:**
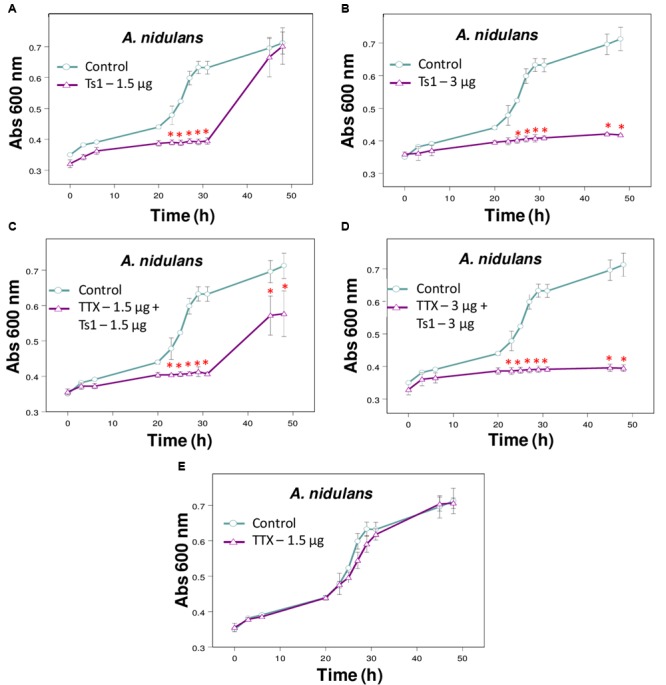
Effect of Ts1 on *A. nidulans* growth in the presence of TTX. Fungal growth was monitored by measuring the absorbance of fungal culture at 600 nm during 48 h, at different intervals of time. Control (open circle, no toxin), Toxins (open triangle). **(A)** Ts1, 1.5 μg/well; **(B)** Ts1, 3 μg/well; **(C)** TTX, 1.5 μg/well + Ts1, 1.5 μg/well; **(D)** TTX, 3 μg/well + Ts1, 3 μg/well; **(E)** TTX, 1.5 μg/well. Each point represents the mean ± SD (*n* = 4) of the absorbance of fungal culture at each time. ^∗^*p* < 0.01 compared to the control (one-way ANOVA). **(C,D)** TTX was added to the medium with fungus at zero time and incubated during 30 min. After that, Ts1 was added.

All experiments were carried out in sterile 96-well flat bottom microplates. Each sample dilution was tested in quadruplicate and the assays were performed at least twice on different days. Each well (final volume of 100 μL) contained 80 μL of half-strength potato dextrose broth (12 mg/mL), 10 μL of the spore suspension diluted in sterile distilled water containing approximately 200 spores, and 10 μL of sterile saline in the absence (positive control) or presence of Tsv (100 and 500 μg, which correspond to 1 and 5 mg/mL, respectively, of total soluble protein) or one of its fractions I-XIIB (3 and 7.5 μg, which correspond to 30 and 75 μg/mL, respectively, of total soluble protein) or Ts1 (1.5, 3, and 6 μg, which correspond to 2.18, 4.36, and 8.72 μM, respectively, since its molecular mass is 6,873 Da). The negative control consisted of the reaction mixture in the absence of toxin and spores. After the inoculation, the microplates were homogenized for a few seconds on a microplate shaker and incubated at room temperature for 30 min prior to the first reading. The toxins (Tsv, its fractions and Ts1) were added to the medium at time zero. For the assay against *A. nidulans*, Ts1 was added to the medium at time zero and after 6 h of growth. The absorbance of each well was measured at 600 nm by a 96-well microplate reader (Sunrise, Tecan Inc., Mannedorf, Switzerland) during 48 h, at different intervals of time (shown in the **Figures 1**–**5**). The data are expressed as mean ± standard deviation (SD) and were analyzed using one-way ANOVA. Values of *p* < 0.05 were considered statistically significant.

To evaluate the effect of Ca^2+^ treatment on the growth of the fungi *A. terreus, P. corylophilum* and *P. verrucosum*, the assay was carried out with the toxin Ts1 (3 and 6 μg/well, which correspond to 4.36 and 8.72 μM, respectively) in the absence or presence of 1 mM CaCl_2_. The effect of Ts1 (1.5 and 3 μg/well, which correspond to 2.18 and 4.36 μM, respectively) against *A. nidulans* was also carried out in the absence or presence of TTX (1.5 and 3 μg/well, which correspond to 46.98 and 93.96 μM, respectively, since its molecular mass is 319.27 Da).

To evaluate if the toxin Ts1 is fungistatic or fungicide, all the content of the well after 48 h of incubation of Ts1 (6 μg/well, which correspond to 8.72 μM) with *A. nidulans* (∼200 spores/well) was applied onto plates containing PDA solid medium and incubated at 30°C for 24 h. Then it was observed if there was growth of the fungus.

### Analysis of Fungus Morphology

Saline solution of Ts1 was previously filtered on a 0.22 μm sterile membrane and aliquots (25 and 50 μL) at 0.6 mg/mL of total soluble protein were added to 3.2 cm-internal diameter sterile Petri plates. The PDA medium (1 mL) at 45°C was added over the toxin solution, homogenized with a sterile 1 mL-tip and allowed to solidify for about 30 min, resulting at approximately 15 and 30 μg/mL (2.18 and 4.36 μM) of Ts1 per plate. The fungus which was more significantly inhibited by Ts1 had approximately 200 spores (10 μL) inoculated on a fragment of sterile dialysis membrane (1.5 cm × 1.5 cm), overlaying PDA medium with toxin. Control group had no toxin. The plates were incubated at 30°C for 12 h. After this period, the dialysis membranes were carefully removed and the hyphae morphology and spores germination were evaluated under a stereo microscope (Leica Microsystems, Wetzlar, Germany). The experiment was done in duplicate and the images were photographed.

### Hemolytic Assay

Adult healthy male sheep were bled by jugular vein puncture and blood was collected in two volumes of Alsever’s modified solution (Hoffmann and Mayer, 1977), which was used as anticoagulant. All animal handling procedures and protocols were performed according to the National Counsel for animal experimentation recommendations (Conselho Nacional de Controle de Experimentação Animal – CONCEA). Animal use was approved by the Committee for Ethics in Animals Utilization of University of São Paulo (n. 05.1.637.53.6). Sheep blood was centrifuged and the plasma and buffy coat were discarded. Red cells were washed three times in phosphate buffered saline (PBS). The red cells solution was standardized in a spectrophotometer (absorbance at 700 nm adjusted to 0.70–0.75) to contain approximately 1.7 × 10^8^ cells/mL (counted in a Neubauer chamber).

To evaluate the lytic activity of Tsv and Ts1, 60 μL of the standardized red cells solution was incubated with 60 μL of PBS and 30 μL of Tsv (500 μg) or Ts1 (6 μg) at 37°C, for 30 min. Then the samples were cooled in an ice bath, mixed with cold PBS (150 μL) and centrifuged at 480 × *g* for 10 min. The percentage of hemolysis was determined by measuring the absorbance of the supernatant at 412 nm (Little and Rumsby, 1980).

### Ts1 Alignment and Structure Comparison

The primary structure of Ts1 was compared with other AMPs deposited in databanks. The amino acid sequences were retrieved from the Universal Protein Resource Knowledgebase^3^ or from the Protein Data Bank^4^. The amino acid sequences were as follows: Ts1 [PDB: 1NPI], drosomycin [PDB: 1MYN], bactridin-1 [Swiss-Prot: P0CF39] and bactridin-2 [Swiss-Prot: P0CF37]. The alignment and the identity percentage were performed using Clustal Omega website^5^, version 1.2.4 (Sievers et al., 2011).

For protein structure alignment and comparison, the algorithm TM-align (Zhang and Skolnick, 2005) was used. The superposed full-atom structure of the entire chains (without ligands and solvent) of Ts1 and drosomycin was performed by VMD (Visual Molecular Dynamics^6^) (Humphrey et al., 1996). The structures and sequences were compared using MultiSeq tool (Roberts et al., 2006) incorporated in VMD and the structure superposition was generated with the PyMOL Molecular Graphics System (DeLano, 2002).

## Results

### Purification of Ts1

Ts1 was isolated as previously described (Vasconcelos et al., 2005) and represented around 16% of Tsv (Supplementary Figures S1A,B). The symmetry of the peak eluted from C18 reversed-phase column (Supplementary Figure S1B) is an indicative of the homogeneity of the sample, which was later confirmed through PAGE, where Ts1 appeared as a single electrophoretic band (data not shown).

### Antifungal Activity Assay

The present screening evaluated the growth patterns of *A. fumigatus, A. nidulans, A. niger, A. terreus, N. crassa, P. corylophilum, P. ochrochloron, P. verrucosum, P. viridicatum, P. waksmanii* and *T. flavus* in the presence of Tsv (100 and 500 μg/well, which correspond to 1 and 5 mg/mL, respectively, of total soluble protein) and in control condition. The results show a high inhibitory effect of Tsv on the growth of *A. nidulans, A. terreus P. corylophilum*, and *P. verrucosum*, especially in the higher concentration used and at the first 30 h, showing a dose-dependent antifungal activity (**Figures 1A–D**). On the other hand, Tsv increased the growth of *N. crassa*, and *P. waksmanii*, but had no effect on the other analyzed fungi (*A. fumigatus, P. ochrochloron, P. viridicatum* and *T. flavus*) at the tested doses (100 and 500 μg/well, which correspond to 1 and 5 mg/mL, respectively, of total soluble protein; data not shown).

The fractions isolated from Tsv (Supplementary Figure S1A) were tested against the same wide range of fungi species. Fractions XI and XIIB (3 μg/well, which correspond to 30 μg/mL of total soluble protein) were active against *A. terreus* (**Figures 2A,B**). Fractions X and XIIA (7.5 μg/well, which correspond to 75 μg/mL of total soluble protein), IX and XIIB (3 and 7.5 μg/well, which correspond to 30 and 75 μg/mL, respectively, of total soluble protein) showed antifungal activity against *A. nidulans* (**Figures 2C–H**).

Ts1 shows a dose-dependent inhibition on the growth of *A. terreus, P. corylophilum* and *P. verrucosum* (**Figures 3A–F**). The addition of 1mM CaCl_2_ did not alter the effect of Ts1 on the growth of *A. terreus* (**Figures 3A,B**), but significantly potentiated the inhibition effect of Ts1on the growth of *P. verrucosum* (**Figures 3C,D**) and of *P. corylophilum* (**Figures 3E,F**).

**Figure 4** shows the effects of Ts1 on the growth of *A. nidulans* on the different tested concentrations (1.5, 3 and 6 μg/well, which correspond to 2.18, 4.36, and 8.72 μM, respectively) and on the different times of spores’ germination (0 and 6 h). Ts1 was able to inhibit the fungal growth in a dose-dependent manner (**Figures 4A–F**) into 96-well flat bottom microplates, showing 100% inhibition from 3 μg/well (4.36 μM). The inhibitory effect of Ts1 (1.5 μg/well, which correspond to 2.18 μM) was statistically significant when the toxin was added to the medium with fungus spores at zero time and incubated during 24 h (**Figure 4A**). Ts1 (6 μg/well, which correspond to 8.72 μM) exhibits a fungistatic activity against *A. nidulans*, since we have observed viable spores on the content of the well after incubation with the toxin (data not shown).

The addition of TTX did not change significantly the inhibitory effect of Ts1 on the growth of *A. nidulans* (**Figures 5A–E**).

### Analysis of Fungus Morphology

The toxin Ts1 was able to reduce the elongation of hyphae from *A. nidulans* in a dose-dependent manner, without causing morphological alterations (Supplementary Figure S2).

### Hemolytic Assay

*Tityus serrulatus* venom and its major toxin Ts1 showed to be non-cytolytic on erythrocytes, even with the higher tested doses (500 and 6 μg, which correspond to 5 mg/mL and 8.72 μM, respectively; data not shown).

### Ts1 Alignment and Structure Comparison

**FIGURE 6:**
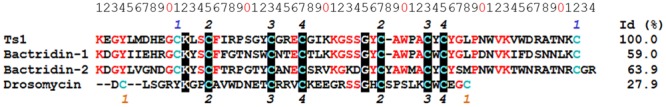
Multiple sequence alignment of Ts1 and AMPs. The alignment and identity (%) of Ts1 [PDB: 1NPI], drosomycin [PDB: 1MYN], bactridin-1 [Swiss-Prot: P0CF39] and bactridin-2 [Swiss-Prot: P0CF37] were performed using Clustal Omega version 1.2.4. The conserved amino acid residues are in red, and those highly conserved are highlighted in black. Cys residues are in blue. The numbers on the top and on the bottom refer to the disulfide bonds – the common bonds are in black; the orange and blue numbers represent the distinct bonds among drosomycin and the other toxins, respectively.

**FIGURE 7:**
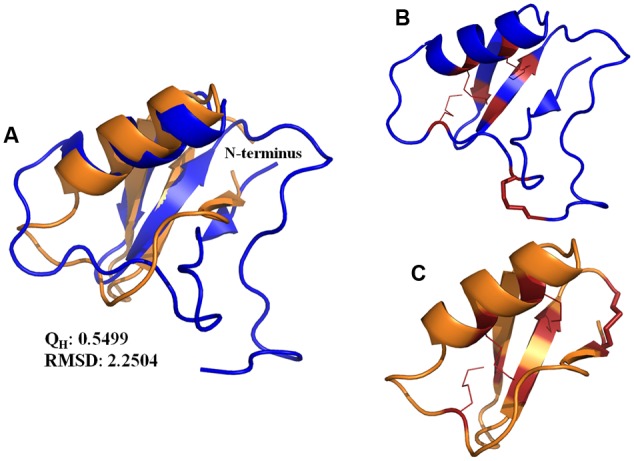
**(A)** Structure superposition of the Ts1 (blue) and drosomycin (orange) chains. The cartoon representation was generated with PyMOL. The Q_H_ and RMSD values were calculated by VMD. **(B,C)** Disulfide bonds are shown as red lines. The stick representation indicates the disulfide bonds in the **(B)** Ts1 and **(C)** drosomycin structures.

Ts1 shares the highest identity score (63.9%) with bactridin-2 (**Figure 6**). Although the percent identity with drosomycin is low (27.9%, **Figure 6**), the *Q*_H_ value of the superposed Ts1 and drosomycin structures is above 0.5 (**Figure 7A**). The Q_H_ measures structural conservation (Eastwood et al., 2001). When Q_H_ has a low score (0.1–0.3), structures are not aligned well. The TM-score = 0.50184 (data not shown) was normalized by length of Ts1 chain using TM-align. TM-score below 0.3 shows random structural similarity (Zhang and Skolnick, 2005). The disulphide bonds are shown in the Ts1 (**Figure 7B**) and drosomycin (**Figure 7C**) structures.

## Discussion

Searches for new antibacterial and antifungal peptides have attracted the attention of many researches in recent years. This excitement is due to the rush in finding new therapeutic agents to deal with the emerging microbial resistance (Pimenta and De Lima, 2005). The spotlights have been pointed toward the AMPs and the use of those molecules as models of unique scaffolds for the development of new antibiotics drugs (Harrison et al., 2014). AMPs represent an ancient defense mechanism against invasive pathogens and are present in several groups of animals. These peptides have been reported as one of the many components present in the complex mixture of scorpion venoms (Harrison et al., 2014).

The antifungal screening performed in the present work showed the efficiency of Tsv in reducing the growth of *A. nidulans, A. terreus, P. verrucosum* and *P. corylophilum* at the first 30 h (**Figures 1A–D**). On the other hand, Tsv increased the growth of the fungi *N. crassa* and *P. waksmanii* (data not shown). Since scorpion venoms are composed by a complex mixture of mucus, low molecular mass molecules and many other peptides and proteins, those species of fungi might have used these components as alternative sources of nutrients, and therefore, increased their growth tax.

The fractions I-XIIB isolated from Tsv were also tested against those 11 fungi species. Fractions XI and XIIB were effective against *A. terreus* only in the lowest tested dose after 48 h of incubation. At higher doses, some venom components may be exerting different and antagonistic effects which could be affecting the fractions’ inhibitory activity. Fractions IX, X, XIIA and XIIB reduced the growth of *A. nidulans*. Ts fractions had no effect on the other fungi (*P. corylophilum* and *P. verrucosum*) affected by the whole venom, maybe because they were tested at doses below the effective dose able to cause inhibition.

*Tityus serrulatus* venom fractionation procedure as described by Arantes et al. (1989) directly yields the toxin Ts1 in the fraction XIII, whose purity was confirmed by reversed-phase chromatography on a C18 column and PAGE. Ts1, also known as TsTX-I, toxin-γ, Toxin T_2_IV, Toxin Ts7, Ts VII, Toxin VII, TsTX-VII, Tityustoxin VII, Toxin III-10, Toxin II-11, or Toxin T_1_VIII (Bordon et al., 2015), is the most abundant and potent toxin from the venom of the Brazilian yellow scorpion *T. serrulatus* and was the first biochemically characterized toxin of this arthropod (Possani et al., 1977; Arantes et al., 1989). Because of the relatively high amount of this toxin on Tsv, accounting for approximately 16% of the soluble venom and the high toxicity against both mammals and insects, many studies concerning Ts1 have been carried out over the years (Cologna et al., 2009; Bordon et al., 2015). Recently, a gamut of effects on Na^+^ channels (Peigneur et al., 2015) and an important immunomodulatory activity on macrophages have been reported for Ts1 (Petricevich et al., 2007; Zoccal et al., 2011, 2014).

Despite the number of studies which investigated Ts1, the present work describes for the first time the antifungal screening using this well known toxin. On the past years, excepting the K^+^ channels blockers toxins which are related to defensins, most of the AMPs described from scorpion venoms were characterized as linear amphipathic peptides (Bulet et al., 2004). However, the identification of the Bactridines in 2009 (Diaz et al., 2009) has changed this concept.

In addition to the antimicrobial activity, bactridines, similarly to Ts1 with which share approximately 60% of sequence identity, showed effect on Na^+^ channels and were classified as β-modulators of these channels. Bactridines were reported to change the bacterial membrane sodium permeability (Diaz et al., 2009). However, the electrophysiological studies revealed no effect of those toxins on the oocyte-expressed NaChBac channel, suggesting they may act on others isoforms of Na^+^ channels, or even in other sodium translocation pathways different from ion channels (Diaz et al., 2009; Peigneur et al., 2012). Equivalently, Ts1 also did not present affinity for NaChBac channel (Peigneur et al., 2015). It is noteworthy that bactridines were the first scorpion toxins presenting antibacterial activity and action on Na^+^ channels (Peigneur et al., 2012), while Ts1 is the first Na^+^ channel toxin with antifungal activity (this work).

Regarding the antifungal effect of Ts1 against *P. corylophilum* and *P. verrucosum*, the activity was strikingly potentiated by the addition of 1 mM CaCl_2_. Adversely, it was reported that the supplementation with cations on test medium does not increase, but does reduce the antifungal activity of defensins. Plant, insect and mammalian defensins had their antimicrobial activity reduced in the presence of divalent cations, especially Ca^2+^ (Lehrer et al., 1988; Cociancich et al., 1993; Osborn et al., 1995). Results previously reported indicate that a direct interaction occurs between the fungus and the cations, which protects the fungus against the inhibitory effect of the protein without conformational changes (Terras et al., 1992). Ionic strength antagonism is dependent on the fungus and the conformation of the putative target site (Terras et al., 1992; Osborn et al., 1995). For instance, the growth medium supplemented with 5 mM CaCl_2_ caused a 1.7-fold reduction of inhibitory activity of Rs-AFP2 (an antifungal protein from radish seeds) against *Fusarium culmorum* and more than 50-fold reduction against *Trichoderma hamatum* (Terras et al., 1992). The offbeat activity of Ts1 against those two fungi still need to be further explored.

Concerning the inhibitory effect of Ts1 against *A. nidulans*, this event was accompanied by fungistatic effects and was not disturbed significantly by the addition of TTX at all tested conditions. TTX is a potent Na^+^ channel blocker (Narahashi et al., 1964), for which there is no known antidote (Bane et al., 2014). In this way, these results indicate that the antifungal activity of Ts1 is not related to its interaction with voltage-gated Na^+^ channels. Fungal genomes contain genes encoding K^+^, Ca^2+^ and transient receptor potential (Trp) channels, but not voltage-gated Na^+^ channels or ligand-gated channels (Prole and Taylor, 2012). However, fungal calcium channels were reported to be closed related to sodium leak channels, non-selective (NALCN) (Liebeskind et al., 2012). Those newly described channels allow the passive flow of sodium (and other cations) across the cell membrane of neurons, modulating the neuronal resting potential (Ghezzi et al., 2014) and working as a sensor that indirectly affects membrane permeability (Senatore et al., 2013). The effects of Ts1 on these channels were never described so far and therefore NALCN could represent a crosshair to elucidate the mechanism of action of this antifungal toxin. Thus, further studies to unveil this conundrum are still required. Nevertheless, based on our results, we can already stress that the described antifungal activity of Ts1 is not related to its action on voltage-gated Na^+^ channels. In addition, our results suggest that some antifungal peptides, such as Ts1, may play their role by different modes-of-action, which might be related to uncharted receptors or other little-known channel types present on the fungus membrane. As a matter of fact, five toxins (F2 to F6) isolated from *T. discrepans* scorpion venom, with masses between 6,924.6 and 7328.8 Da, typical of scorpion toxins acting on sodium channels, showed antifungal effects against the phytopathogenic fungus *Macrophomina phaseolina* through three different ways: inhibiting fungus esterases and altering Na^+^ membrane permeability and/or sterol biosynthesis (Joya et al., 2011). In this way, in-depth studies regarding Ts1 antifungal activity are necessary, in order to answer further questions, like its mechanism of action and its respective MIC or minimum effective concentration (MEC) quality control (QC) limits.

Another issue to be considered is that Ts1 is a cationic peptide [pI ∼ 8.67 (Cologna et al., 2009)]. Cationic peptides may destabilize membranes at a given concentration (Remijsen et al., 2010), but the mechanism of action of some of them is still unclear (Jenssen et al., 2006; Mookherjee and Hancock, 2007). Parabutoporin is an example of scorpion cationic amphipathic lysine-rich and cysteine-free peptide with antimicrobial, antifungal and immunoregulatory activities (Remijsen et al., 2010). *In silico* and circular dichroism analyses of seven antifungal NDBPs highlighted the relevance of cationicity and amphipathicity for their antimicrobial activity (Guilhelmelli et al., 2016). AamAP1-Lysine, a synthetic peptide analog of a scorpion AMP, was modified to increase the positive charge from the native peptide, resulting in bactericidal activity through membrane damage and permeabilization caused by β-galactosidase releasing from bacterial cells (Almaaytah et al., 2014). Designed cationicity-enhanced analogs of natural AMPs from *Androctonus crassicauda, A. aeneas, Vaejovis mexicanus smithi, U. yaschenkoi* and *U. manicatus* scorpion venoms exhibited higher potency and spectra of antimicrobial activity (Du et al., 2014, 2015; Luna-Ramirez et al., 2017; Pedron et al., 2017).

Regarding the structural features of Ts1, its primary structure is composed of 61 amino acid residues with a molecular mass of 6,873 Da (Pinheiro et al., 2003). The tertiary structure of this toxin has been determined by X-Ray crystallography and shows a conserved core formed by three antiparallel β-strands and an α-helix stabilized by four disulfide bonds (Polikarpov et al., 1999). This conserved motif is known as Cysteine Stabilized αβ (CSαβ) motif and is also found in defense peptides exhibiting antibacterial and antifungal activities, such as insect defensin A, plant thionins and plant defensins (Landon et al., 1997). The deletion of a small loop of the defensin structure resulted in a K^+^ channel blocking neurotoxin, providing evidences that K^+^ channel toxins and defensins are evolutionarily related (Zhu et al., 2014).

In an attempt to establish a structure-activity relationship for the antifungal effect of Ts1, we performed the structure alignment of Ts1 and drosomycin, a potent cysteine-rich antifungal peptide. Multiple sequence structure alignment reveals much important information, e.g., proteins with similar functions tend to be found with similar structural features. Ts1 and drosomycin structures displayed good alignment, as shown by the Q_H_ and RMSD (root-mean-square distance) values. Additionally, the TM-score above 0.5 indicates that Ts1 and drosomycin have the same fold. Although few amino acid residues are conserved between Ts1 and drosomycin (27.9% of sequence identity), both of them share the same structural CSαβ scaffold. Ts1 structure shows a longer *C*-terminal loop than drosomycin and a disulfide bond with distinct position linking the *C*- and *N*-domains.

Drosomycin has a potent antifungal activity while it is ineffective against yeasts and bacteria and has no hemolytic effect on bovine erythrocytes (Bulet et al., 1999). Concerning the mode-of-action, the distinctive activity of drosomycin on *N. crassa* and its mutant indicated a possible interaction of the peptide with fungal sphingolipid receptors, resulting in fungal cell death due to membrane permeabilization (Gao and Zhu, 2008; Wilmes et al., 2011).

The fungicidal activity of drosomycin against *N. crassa* has been reported and has shown that this compound is a morphogenic peptide (Fehlbaum et al., 1994). Morphogenic defensins slow down hyphal elongation associated to an increase in hyphal branching, whereas non-morphogenic defensins reduce hyphal extension with no pronounced morphological distortions (Osborn et al., 1995; Aerts et al., 2008).

Based on mutation or deletion assays, it has been proposed that the effects observed on hyphae morphology may be explained by the presence or absence of hydrophobic residues in the turn connecting strands II and III of CSαβ proteins (DeSamblanx et al., 1997; Cohen et al., 2009). Non-morphogenic proteins contain polar residues in this interstrand loop, while morphogenic ones exhibit a hydrophobic cluster at the protein surface in which a lysine residue is embedded (Cohen et al., 2009). Cohen et al. proposed that the antifungal active site of drosomycin is formed by the hydrophobic cluster Leu3, Pro10, Pro35, Leu37 and Trp40 where the basic residue Lys38 is embedded (Cohen et al., 2009). On Ts1, these residues correspond to Asp7, Ser14, Ala38, Pro40, Tyr43 and Ala41. These differences could justify why Ts1 does not alter the morphology of the treated hyphae. Additionally, we can conclude that Ts1 causes no interference at morphogenetic Ca^2+^ signaling, since the branching of fungal hyphae is regulated by specific Ca^2+^ channels (Robson et al., 1991).

In respect to electrostatic interactions, drosomycin caused partial lysis of the growing hyphae of *Botrytis cinerea* in half-strength potato dextrose broth supplemented with 1 mM CaCl_2_ and 20 mM KCl (Fehlbaum et al., 1994). Monovalent and especially divalent cations in the medium play an antagonizing effect on defensins’ antimicrobial activity (for review, see Aerts et al., 2008). This antagonistic effect depends on the tested fungus, indicating that electrostatic interactions on the fungal membrane are relevant for the antifungal activity (Broekaert et al., 1995). As previously mentioned, the addition of CaCl_2_ potentiated the inhibitory effect of Ts1, reinforcing that Ts1 may present a possible new antifungal mode-of-action.

By virtue of the increasing antimycotic drug resistance, the discovery of new mechanisms of action with greater antifungal specificity can lead to a potential therapeutic innovation.

## Conclusion

*Tityus serrulatus* venom, its fractions IX, X, XI, XIIA, XIIB and its major toxin Ts1 inhibited fungi growth. Ts1 is a voltage-gated Na^+^ channel toxin that may be performing a striking antifungal mode-of-action, since voltage-gated Na^+^ channels have not been reported in fungi. Further studies to enlighten its mechanism of action on fungi are necessary. Ts1 is a multifunctional toxin which may be a relevant research tool in the design of engineered scorpion AMPs and in the search for new mechanisms of action of antifungal drugs.

## Author Contributions

WS and ARA carried out all the experiments. CC helped in drafting the manuscript. KB performed the structural alignment and drafted the manuscript. SS participated in the design of the antifungal assays. EA is the corresponding author and designer of the research. WS and KB contributed equally to this work. All authors analyzed the results, revised the manuscript and approved the final version.

## Conflict of Interest Statement

The authors declare that the research was conducted in the absence of any commercial or financial relationships that could be construed as a potential conflict of interest.

## Footnotes

1 http://web.expasy.org/cgi-bin/protparam/protparam

2 http://fat.org.br/

3 www.uniprot.org

4 http://www.rcsb.org/pdb/home/home.do

5 http://www.ebi.ac.uk/Tools/msa/clustalo/

6 http://www.ks.uiuc.edu/Research/vmd/

## Abbreviations

*A.*: *Aspergillus*
AMPs: antimicrobial peptides
*N*.: *Neurospora*
NDBPs: non-disulfide-bridged peptides
*P.*: *Penicillium*
PDA: potato dextrose agar
*T.*: *Talaromyces*
Tsv: *Tityus serrulatus* venom
TTX: tetrodotoxin
