# Supplementary material for: Antifungal Activity against Filamentous Fungi of Ts1, a Multifunctional Toxin from Tityus serrulatus Scorpion Venom
Source: Front Microbiol. 2017 Jun 6;8:984. doi: 10.3389/fmicb.2017.00984 (PMC5459920; doi:10.3389/fmicb.2017.00984)
Supplement: Supplementary file 1 [file Data_Sheet_1.pdf]

Supplementary Material

Antifungal Activity Against Filamentous Fungi of Ts1, a Multifunctional Toxin from *Tityus serrulatus* Scorpion Venom

Welligton de Mattos Santussi[1§], Karla de Castro Figueiredo Bordon[1§], Ana Paula Nunes Rodrigues Alves[1], Camila Takeno Cologna[1], Suraia Said[2], Eliane Candiani Arantes[1*]

[1]Laboratory of Animal Toxins, Department of Physics and Chemistry, School of Pharmaceutical Sciences of Ribeirão Preto, University of São Paulo, Ribeirão Preto, SP, Brazil

[2]Laboratory of Industrial Enzymology, Department of Pharmaceutical Sciences, School of Pharmaceutical Sciences of Ribeirão Preto, University of São Paulo, Ribeirão Preto, SP, Brazil

§ These authors contributed equally to this work

*** Correspondence:** Eliane Candiani Arantes: ecabraga@fcfrp.usp.br

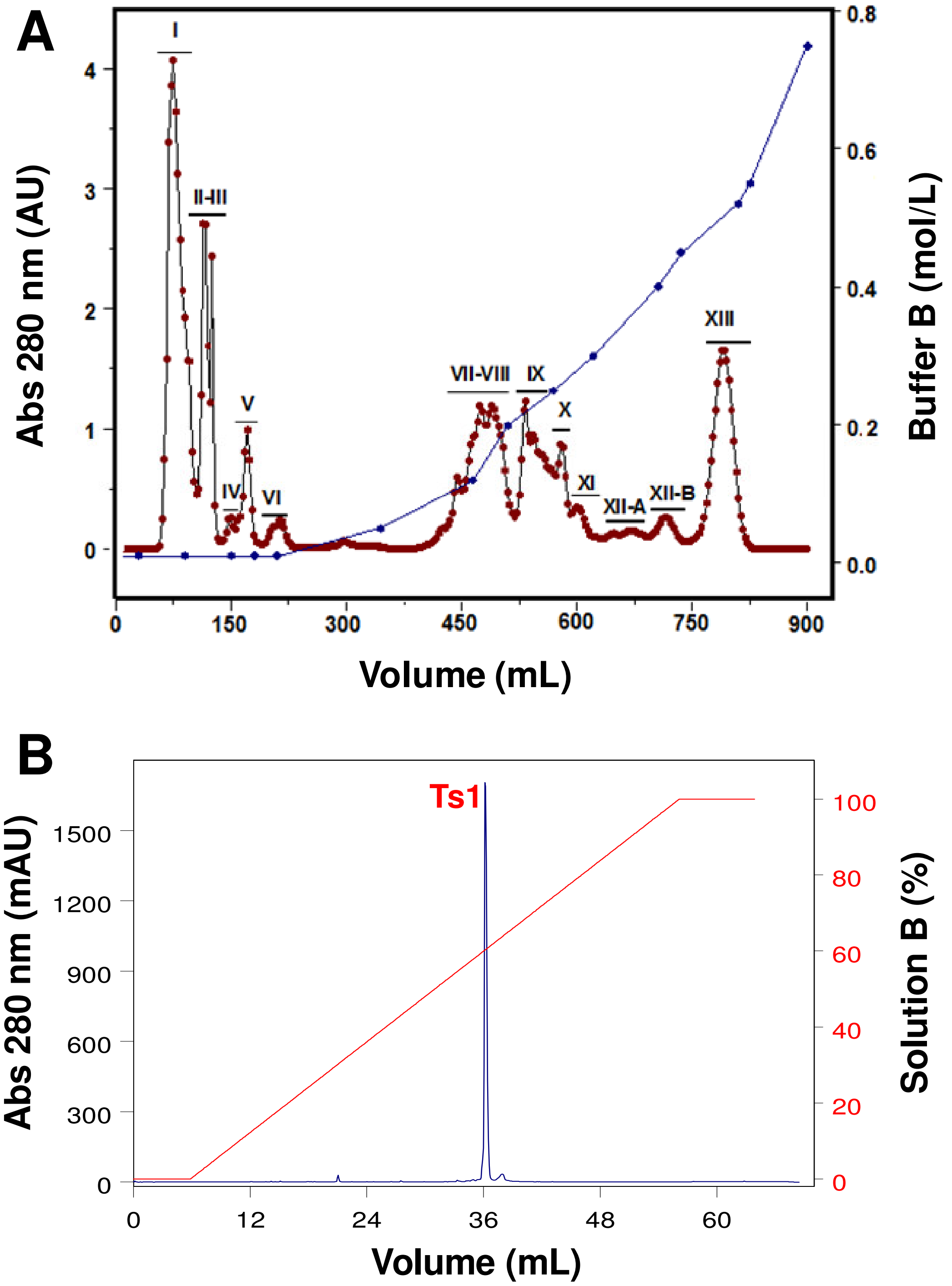

Figure S1. (A) Elution profile of the soluble *T. serrulatus* venom (200 mg) on a CM-cellulose-52 column (2.5 x 67.0 cm). The proteins were eluted using a convex concentration gradient from 0.05 to 1.0 M ammonium bicarbonate, pH 7.8, at a flow rate of 20 mL/h. **(B) Reversed-phase of fraction XIII to obtain Ts1.** The proteins adsorbed to the C18 column (4.6 mm x 250.0 mm, 5 µm particles, 300 Å, Shimadzu) were eluted with a concentration gradient from 0% to 100% of solution B (80% acetonitrile in 0.1% TFA), at a flow rate of 0.8 mL/min.

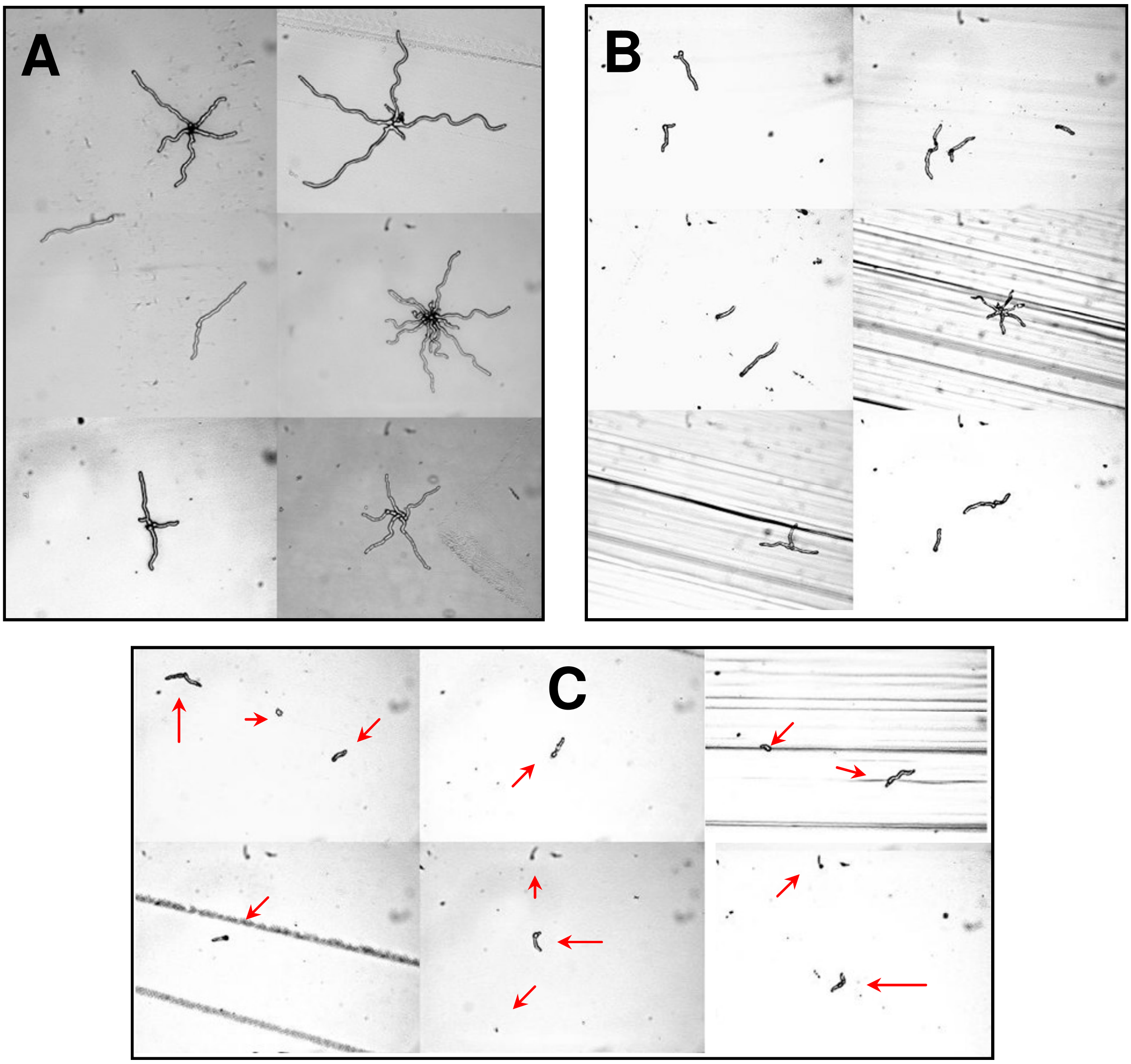

Figure S2. ***Aspergillus nidulans*** **morphology in the absence and presence of Ts1. (A)** control; **(B)** Medium supplemented with Ts1 (15 µg); **(C)** Medium supplemented with Ts1 (30 µg). The photos were taken after 12 hours of fungi growth (increasing 200 x). The arrows in the group "C" indicate spores emitting tube germination and non-germinated spores.
